# The Role of Surgery in the Treatment of Cervical Lymph Node Tuberculosis

**DOI:** 10.7759/cureus.38824

**Published:** 2023-05-10

**Authors:** Ilias Tahiri, Rim Yacoubi, Othman Elhouari, Said Anajar, Taali Loubna, Amal Hajjij, Mohammed Zalagh, Khalid Snoussi, Mustapha Essaadi, Fouad Benariba

**Affiliations:** 1 Otolaryngology - Head and Neck Surgery, Mohamed VI University of Health Sciences/Cheikh Khalifa International University Hospital, Casablanca, MAR; 2 Otolaryngology - Head and Neck Surgery, Mohammed V Military Training Hospital, Rabat, MAR; 3 Otolaryngology - Head and Neck Surgery, Mohamed VI University of Health Sciences/Cheikh Khalifa International University Hospital, Rabat, MAR

**Keywords:** drainage, lymphadenectomy, lymph node dissection, adenectomy, cervicotomy, biopsy, surgery, cervical lymph node tuberculosis

## Abstract

Cervical lymph node tuberculosis is a public health problem in Morocco and the rest of the world. Its paucibacillary nature makes diagnosis and treatment difficult. This is a descriptive-analytical retrospective study presenting 104 cases of patients with manifestations of cervical lymph node tuberculosis confirmed by pathological examination (100%), associated in some cases with positive bacteriology (40.6%), treated and followed up in the otolaryngology (ENT) department of the Cheikh Khalifa International University Hospital (HUICK) over a period of 5 years and 9 months (from January 01, 2017, to September 30, 2022).

In our study, 14 patients (i.e., 13.5%) had a history of tuberculosis (all locations); only four (i.e., 3.8%) of them had confirmed cervical lymph node tuberculosis, of which three were still under treatment: two of them presented for treatment failure (i.e., 1.9%) and one patient for a paradoxical reaction (i.e., 1%). Three pulmonary locations (i.e., 2.9%) and one mediastinal location (i.e., 1%) were found. Surgery associated with histological study was the key to the diagnosis of tuberculosis in our study. Its procedures were: excisional biopsy for 26 patients (i.e., 25%), adenectomy for 54 patients (i.e., 51.9%), lymph node dissection for 15 patients (i.e., 14.4%), and lymphadenectomy for nine patients (i.e., 8.7%). In some cases, drainage (+/- curettage) was recommended in addition to the surgical procedure in 14 patients (i.e., 13.5%). All our patients benefited from post-surgical anti-bacillary treatment. Lymphorrhea was the only operative complication and it affected two patients (i.e., 1.9%). Meanwhile, the relapse rate was 10.6% (i.e., 11 patients), the treatment failure rate was 3.8% (i.e., four patients), and the paradoxical reaction affected 2.9% (i.e., three patients). The latter had all benefited from a simple biopsy. This indicates that a more extensive surgical procedure gives better results with a better healing rate.

In conclusion, anti-bacillary treatment remains the reference treatment for lymph node tuberculosis. However, surgery holds great promise as the first-line treatment in case of fistula or abscess or in the event of failure or if complications occur.

## Introduction

Tuberculosis (TB) is a chronic, infectious, and contagious granulomatous disease in the latent stage, mainly caused by *Mycobacterium tuberculosis* (MTB) [1]. It remains a public health problem worldwide as it is cited among the 10 leading causes of death for adults [2]. The Human Immunodeficiency Virus (HIV) pandemic has played an important role in the resurgence of TB by promoting clusters of nosocomial epidemics of multidrug-resistant TB [1].

At the global population level, more than nine million new cases of TB are reported each year [3,4]. In Morocco, the number of TB cases identified in 2021 was 29,327, which represents an incidence of 81 per 100,000 cases per year [5]. Pulmonary Tuberculosis (PT) accounts for 54% of all locations, while Extra- Pulmonary Tuberculosis (EPT) accounts for 46% [5]. The lymph node is the most common extra-pulmonary location, accounting for 52% of all extra-pulmonary locations [5,6]. The cervical form is predominant, accounting for 70% to 90% of all lymph node locations [5,7]. The diagnosis of TB is based on standard microbiology, but also on histological study. However, molecular biology has revolutionized the means available for the diagnosis of lymph node tuberculosis (LNTB) [8].

The paucibacillary character of LNTB poses a multitude of problems [9,10]. At the diagnostic level, the major problem is that the diagnosis is often carried out at an advanced stage when medical treatment is no longer possible. At the surgical level, the major problem arises due to the risks associated with adhesions, narrow neurovascular relationships, and aesthetic problems. The treatment of LNTB is essentially medical. However, surgery is largely used not only for diagnostic but also for therapeutic purposes even before starting anti-bacillary treatment in certain specific indications, for example in the event of a tubercular cold abscess, or an adenopathy fistulized to the skin [11].

Based on this descriptive-analytical study of 104 cases of LNTB and the review of the relevant literature, we propose an epidemiological and clinical profile of cervical LNTB and also discuss the place of surgery in the therapeutic arsenal.

## Materials and methods

This is a descriptive-analytical retrospective study, conducted in accordance with the Strengthening The Reporting Of Cohort Studies in Surgery (STROCSS) guidelines [12], conducted within the ENT department of the Cheikh Khalifa International University Hospital (HUICK) over a period of 5 years and 9 months and covering 104 patients with manifestations of cervical LNTB and having undergone surgery for diagnostic or therapeutic purposes.

Our work aims to draw up an epidemiological and clinical profile of patients admitted for cervical LNTB requiring surgical treatment and provide an overview of the different surgical techniques offered revealing their processes, advantages, and limitations. Furthermore, we discuss the place of surgery in the therapeutic arsenal for the management of cervical LNTB and finally compare our results with those reported in the literature.

The patients included in our study were patients with cervical LNTB as confirmed by either the presence of an epithelio-giganto-cellular granuloma with or without caseous necrosis on the anatomopathology; or by bacteriology or molecular biology. The patients excluded from our study were the patients with no follow-up after initial treatment, or patients whose records were incomplete.

Informed and written consent was obtained from the participants prior to their inclusion in the study, as well as for the use of any related photographs, while ensuring the protection of their anonymity.

## Results

**Figure 1:**
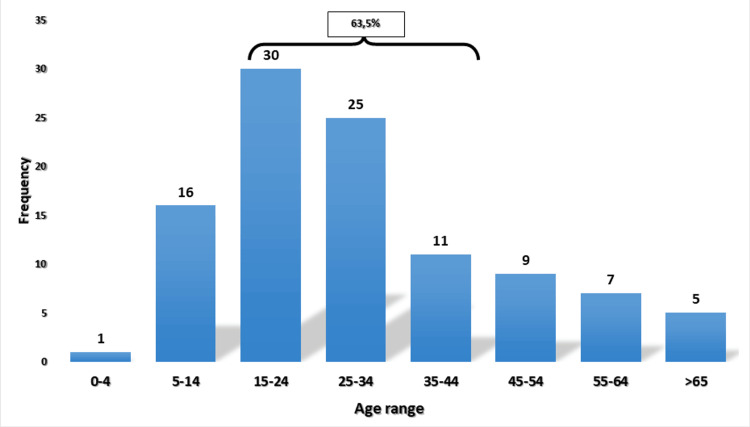
Case distribution by age

One hundred and four (104) cases of cervical LNTB were studied at the ENT department of the HUICK over a period of 5 years and 9 months. Cervical LNTB affects both sexes, with a clear female predominance: 64 women (i.e., 61.5%) for 40 men (i.e., 38.5%). The sex ratio is therefore 1.6:1. The average age of our patients was 30 years with the ages ranging from four years to 78 years. The most affected age groups were those between 15 and 24 years with 30 patients (i.e., 28.8%), between 25 and 34 years with 25 patients (i.e., 24%), and between 5 and 14 years with 16 patients (i.e., 15.4%). The broad age group ranging between 15 and 44 years represented 66 patients (i.e., 63.5%) (Figure 1).

The main factors predisposing to TB in our study were the consumption of unpasteurized milk in 19 patients (i.e., 18.3%) and the notion of TB contamination in 13 patients (i.e., 12.5%). Among the 104 patients in our study, there were 90 new cases of cervical LNTB (i.e., 86.5%), 11 relapses (i.e., 10.6%), two treatment failures (i.e., 1.9%) and one paradoxical reaction (i.e., 1%) (Table 1).

**Table 1:** Distribution by history of tuberculosis LNTB: Lymph node tuberculosis TB: Tuberculosis

Case Type	Number of patients (i.e., %)	Location
New cases	90 (86.5%)	LNTB
Relapse	11 (10.6%)	7 Pulmonary TB
		2 Digestive TB
		1 Mammary TB
		1 LNTB
Treatment failure	2 (1.9%)	LNTB
Paradoxical reaction	1 (1%)	LNTB
Total	104 (100%)	

Cervical swelling was the main reason for consultation and was reported for 100 patients (i.e., 96.2%), while the cervical fistula only concerned four patients (i.e., 3.8%). Cervical pain was not reported alone in any of our patients. However, it was associated with cervical swelling or with fistula in 19 patients (i.e., 18.3%) (Table 2).

**Table 2:** Classification by reason for consultation

Symptom	Number of patients (i.e., %)	Number of patients with cervical pain associated
Cervical swelling	100 (96.2%)	17
Cervical fistula	4 (3.8%)	2
Total	104 (100%)	19

Of the 17 patients for whom the TB skin test was performed, only three patients presented a negative tuberculin intradermal reaction (IDR). A cervical ultrasound was requested for 92 of the patients (i.e., 88.5%) and the results showed that 70 of the patients (i.e., 76.1%) had hypo-echoic appearance, 22 patients (i.e., 23.9%) had hyper-echoic appearance, and 38 patients (i.e., 41.3%) had an aspect of necrosis. Histologic examination was carried out in all our patients, which revealed the presence of an epithelio-giganto-cellular granuloma with or without caseous necrosis. Bacteriological testing for MTB was carried out for 64 patients (i.e., 61.5%), either by testing for Acid-Fast Bacilli (AFB) via direct examination and culture (49 patients, i.e., 76.6%), or by testing for AFB in sputum (four patients, i.e., 6.2%), or by polymerase chain reaction (PCR) via the GeneXpert (Cepheid, Sunnyvale, USA) (11 patients, i.e., 17.2%). In total, TB was revealed in 26 patients, (i.e., 40.6%) (Table 3).

**Table 3:** Bacteriological confirmation of tuberculosis TB: Tuberculosis; AFB: Acid-fast bacilli; PCR: Polymerase chain reaction

Bacteriological testing	Number of patients	TB confirmed
AFB (direct examination and culture)	49	12 (24.5%)
AFB sputum	4	3 (75%)
PCR (GeneXpert)	11	11 (100%)
Total	64	26 (40.6%)

In our study of 104 patients, 90 (i.e., 86.5%) were new cases who received anti-bacillary treatment based on a classic 2RHZE/4RH regimen; the remaining 14 patients (i.e., 13.5%) already had a history of TB either treated or were undergoing treatment according to the Moroccan National TB control program guidelines.

**Figure 2:**
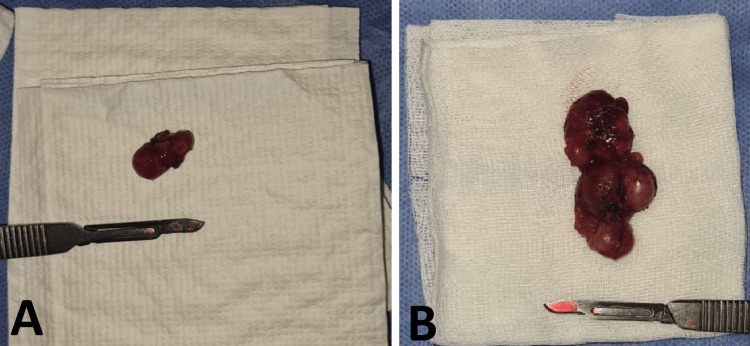
Surgical specimen A : Specimen of single large adenopathy B : Specimen of polyadenopathy Photos from Cheikh Khalifa International University Hospital ENT department.

Surgical treatment was offered to patients in our study for the following indications: single large adenopathy (greater than 30 mm) for 52 patients (i.e., 50% ) (Figure 2A); magma of lymphadenopathy or rosary appearance for 10 patients (i.e., 9.6%) and 13 patients (i.e., 12.5%), respectively; polyadenopathy for 11 patients (i.e., 10.6%) (Figure 2B); and fistulization for four patients (i.e., 3.8%). Cold abscesses were found during surgery in 14 patients (i.e., 13.5%) requiring the addition of a drainage with the initial surgical procedure (Table 4).

**Table 4:** Indications for surgery

Surgical treatment	Number of patients (i.e., %)
Single large adenopathy	52 (50%)
Magma of lymphadenopathy	10 (9.6%)
Rosary appearance	13 (12.5%)
Polyadenopathy	11 (10.6%)
Fistulization	4 (3.8%)
Relapse or paradoxical reaction or therapeutic failure	14 (13.5%)
Total	104 (100%)

All our patients were admitted for an exploratory cervicotomy for diagnostic and/or therapeutic purposes. Gestures performed were mainly the following: simple adenectomy in 51.9% of cases (i.e., 54 patients); biopsy - subtotal excision in 25% of cases (i.e., 26 patients); lymph node dissection in 14.4% of cases (i.e., 15 patients); lymphadenectomy in 8.7% of cases (i.e., nine patients). The drainage (+/- curettage) was performed, in association with another surgical procedure when the surgeon found an abscess, in 13.5% of cases, (i.e., 14 patients) (Table 5).

**Table 5:** Operating techniques performed

Gesture	Number of patients (i.e., %)
Simple adenectomy	54 (51.9%)
Biopsy - subtotal excision	26 (25%)
Lymph node dissection	15 (14.4%)
Lymphadenectomy	9 (8.7%)
Total	104 (100%)

**Figure 3:**
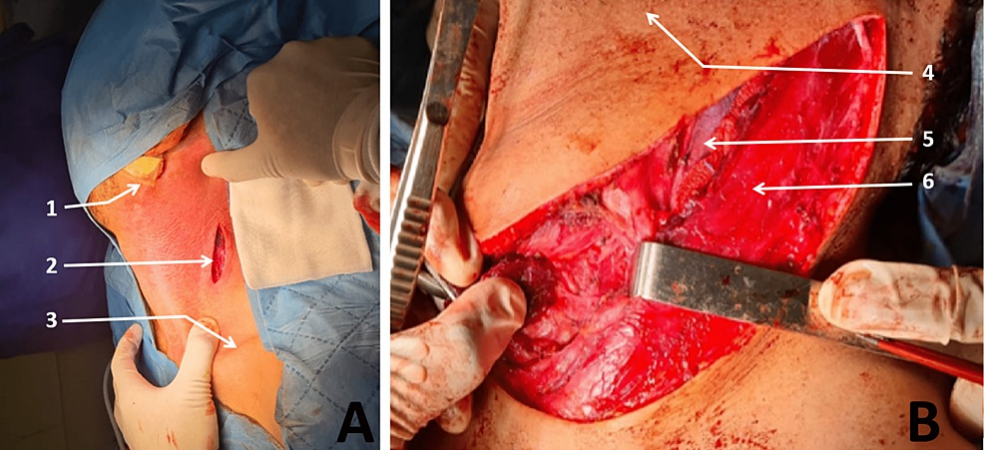
Types of incisions A: Arciform incision centered on the adenopathy 1: Earlobe 2: Incision 3: Clavicle B: Incision of Paul-Andre 4: Chin 5: Internal jugular vein 6: Sternocleidomastoid muscle Photos from Cheikh Khalifa International University Hospital ENT department

LNTB surgery has some limitations due to possible adhesions following the inflammation induced by the TB itself. As a result, different types of incisions have been made by the ENT surgery team, among them: the centered arcuate incision was used in more than 55.8% of cases (i.e., 58 patients) (Figure 3A); and the Paul-André incision was performed in 13.5% of cases (i.e., 14 patients) (Figure 3B).

**Figure 4:**
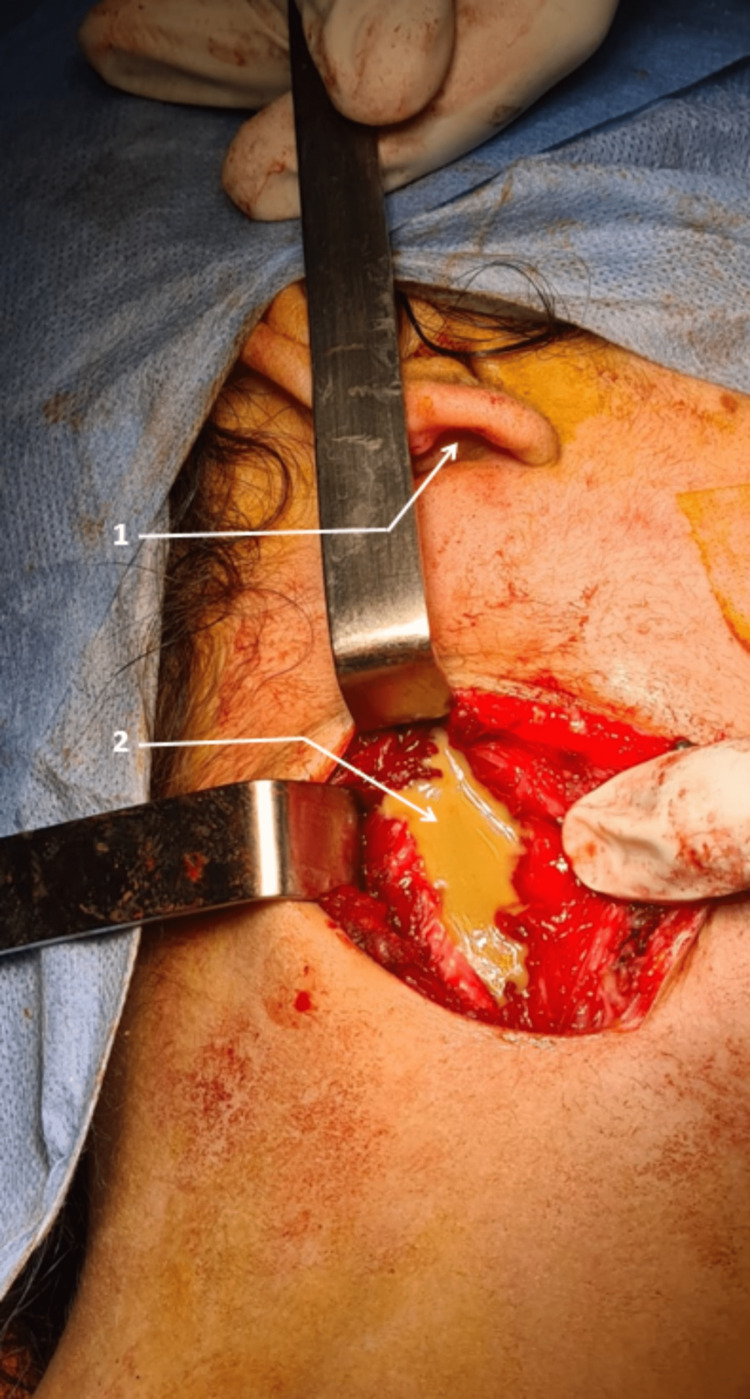
Intra-operative aspect before the drainage of a cold abscess 1: Earlobe 2: Pus Photo from Cheikh Khalifa International University Hospital ENT department

During the intervention, the appearance was strongly suggestive of TB according to the surgeon in 54.8% of cases (i.e., in 57 patients). The main remarks (with an overlap in some cases) of the surgeons included the presence of: caseum in 52 patients (i.e., 50%), inflammatory aspect in 46 patients (i.e., 44%); pus in 30 of them (i.e., 28%) (Figure 4); adhesions to surrounding tissues in 25 patients (i.e., 24%); and a high vascular aspect, bleeding on contact in seven patients (i.e., 6.7%) (Table 6).

**Table 6:** Intra-operative aspect

Aspect	Number of patients (i.e., %)
Caseum	52 (50%)
Inflammation	46 (44%)
Pus	30 (28%)
Adhesions to surrounding tissues	25 (24%)
High vascularity	7 (6.7%)

In our study, 102 of our patients had a straightforward post-operative course without significant complications. Only two patients (i.e., 1.9%) presented lymphorrhea, inducing their return to the operating room for draining on day 3 post-operative for the first patient and day 8 post-operative for the second. Meanwhile, the relapse rate was 10.6% (i.e., 11 patients), the treatment failure rate was 3.8% (i.e., four patients), while the paradoxical reaction only affected 2.9% (i.e., three patients) (Table 7).

**Table 7:** Evolution of our patients

Cases	Number of patients	Evolution		
		Relapse	Treatment failure	Paradoxical reaction
New cases	90	0	2	2
Old cases	14	11	2	1
Total	104	11 (10.6%)	4 (3.8%)	3(2.9%)

## Discussion

In our study comprising 104 patients, the average age was 30 years. The data in the literature points in the same direction with an average age ranging from 29 years [13] to 36 years [14]. The most affected age group was between 15 and 44 years, with 63.5% of cases (i.e., 66 patients). This is in line with the epidemiological data of Morocco for the year 2021 with a rate of 61% for the same age range [5]. The rest of the epidemiological data is similar to the data in the literature with a predominance of the female gender [15,16], all in a population mainly vaccinated with Bacillus Calmette-Guérin (BCG) [17].

In the literature, the notion of TB infection is reported in 10% to 25% of cases [4,18]. This agrees with the results of our study with a rate of 12.5%. Unlike two studies that reported 26.6% [13] and 3% [14]. A history of TB was found in 13.5% of our patients, lower than the rate found in the literature: 26% [13]. The ingestion of unpasteurized milk represents a significant risk of zoonotic transmission [19]. In our study, the consumption rate of unpasteurized milk represents 18.3% of all our patients. This is similar to both Moroccan [13] and Tunisian [20] studies with 14.3% and 25.5% respectively. While another study reports a high consumption rate of unpasteurized milk of up to 90% [14].

LNTB is characterized by an insidious onset and presents in the form of swelling, most often unilateral, non-painful, and chronic course of singular or multiple adenopathies [21,22]. In our study, the main reason for consultation was cervical swelling in 100 patients (i.e., 96.2%). This is similar to the data in the literature with rates ranging from 74% [23] up to 100% [24]. According to our results, consultation time varied between 3 weeks and 7 months, which is consistent with most studies [24,25]. On the other hand, another study reported a longer delay of up to 9 months instead of 7 [26].

TB can affect all lymph node areas. However, the jugulo-carotid chain was the most affected in several studies [27], followed by the spinal chain and then the supraclavicular chain. This same observation was made in our study. Only one study did not follow this pattern, reporting a predominance of the supraclavicular seat in 44% of cases, followed by the jugulo-carotid seat in 36% of cases, and finally the spinal seat in 12% of cases [28].

Our data concerning laterality agree with those found in the literature. Those studies showed that the adenopathies were preferentially unilateral with a predominance on the right [28,29]. The average size of lymphadenopathy varies between 2 cm and 4 cm [28,29]. Large adenopathies, that is to say greater than 3 cm, were predominant [28,29]. This same trend was observed in our study with a predominance of adenopathies of more than 3 cm in about 63.5% of cases (i.e., 66 patients). This can be explained by the chronic nature of TB and therefore the delay in consultation and diagnosis. Initially, the adenopathies are firm in nature and tend towards softening until fistulization [30]. In a Tunisian study [31], 69.7% of the adenopathies were firm, 21.2% soft, and 9.1% hard. This agrees with our results with respective rates of 60.5% (i.e., 63 patients), 26% (i.e., 27 patients), and 13.5% (i.e., 14 patients).

Although histology alone is not sufficient to confirm TB [32], the demonstration of an epithelio-giganto-cellular granuloma with caseous necrosis makes it possible to guide the diagnostic and therapeutic approach. However, histology is best combined with a GeneXpert test or bacteriological culture examination. To do this, it is recommended to keep the sample in saline or distilled water during its transport from the operating room to the laboratory. In contrast, for the anatomopathological examination, the surgical specimen must be preserved in formalin [17]. Most authors report a superiority of the search for Koch's bacillus (BK) by culture compared to direct examination [11,13]. In our study, the Acid-Fast Bacilli (AFB) were highlighted in 12.5% of cases and the culture came back positive in 18.7% of cases. However, some authors report more positive direct BK than positive culture BK [33,34]. The differences between these studies can be explained not only by the paucibacillary character of the LNTB, but also by the secondary errors during the sample’s transport [11,17]. Nevertheless, it should be remembered that a negative direct examination or a negative culture does not eliminate the diagnosis of TB.

Fine needle aspiration requires a trained cytologist. Moreover, we found two studies agreeing with the data in the literature and confirming the superiority of exploratory cervicotomy with a sensitivity reaching 90% to 100% compared to fine needle aspiration with a sensitivity of 88% [35,36]. This is the reason for resorting to cervicotomy (in 100% of our patients) rather than cytopuncture (in only 2.9% of patients; i.e., three patients). Indeed, for the three patients who benefited from a cytopuncture, the result was inconclusive because of the highly hemorrhagic character, thus slowing down the visualization of the AFB.

Tuberculosis, whether pulmonary or extra-pulmonary, retains a high morbidity and mortality rate in the world [37]. It remains however a curable infection [38]. The first line of treatment against TB is medical, the goals of which relate to the following aspects [39]: fight against the infectious source(s) by sterilizing them; act quickly to avoid complications; fight against resistant and multi-resistant strains; prevent the subsequent appearance of new resistance in the event of the presence of a resistant strain beforehand; and act appropriately to avoid post-therapeutic relapses. However, medical treatment has its limitations. In a case-control study, the main causes of relapses reported [40] were: poor patient compliance; immunosuppression; high bacillary load; and errors in understanding the therapeutic protocol.

Surgery as part of the therapeutic management of tuberculous lymphadenitis remains a hot topic. However, consensus regarding the optimal approach is currently lacking. The different goals of surgical treatment are first and foremost, to allow confirmation of TB and then to relieve the discomfort related to the compression due to the mass effect of the lymphadenopathy. Finally, to verify the presence or absence of live bacilli in the event of a paradoxical reaction [41].

**Figure 5:**
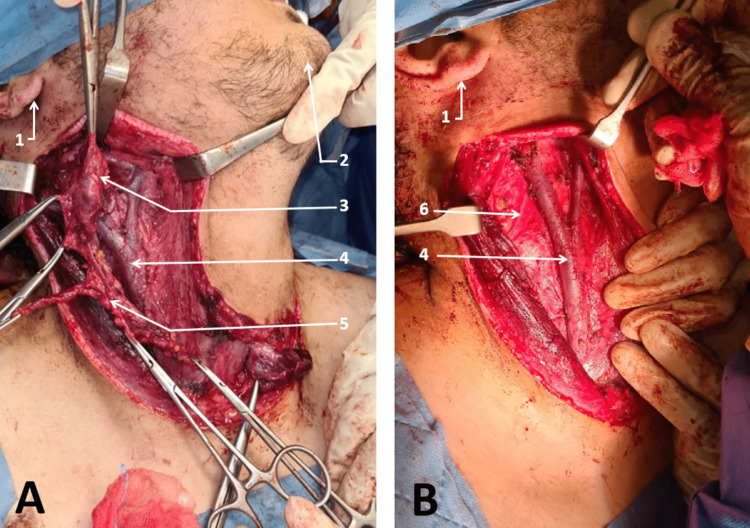
Jugular chain before and after lymph node removal A: Before lymph node removal B: After lymph node removal 1 : Earlobe 2 : Chin 3 : Adenopathy 4 : Internal jugular vein 5 : Chain of adenopathies 6 : Spinal nerve Photos from Cheikh Khalifa International University Hospital ENT department

Concerning the surgical procedures, drainage +/- curettage is indicated in front of a cold abscess or a cutaneous fistula after a puncture which allows bacteriological study [11]. Lymph node dissection varies according to the location of the adenopathies [42] (Figure 5).

**Figure 6:**
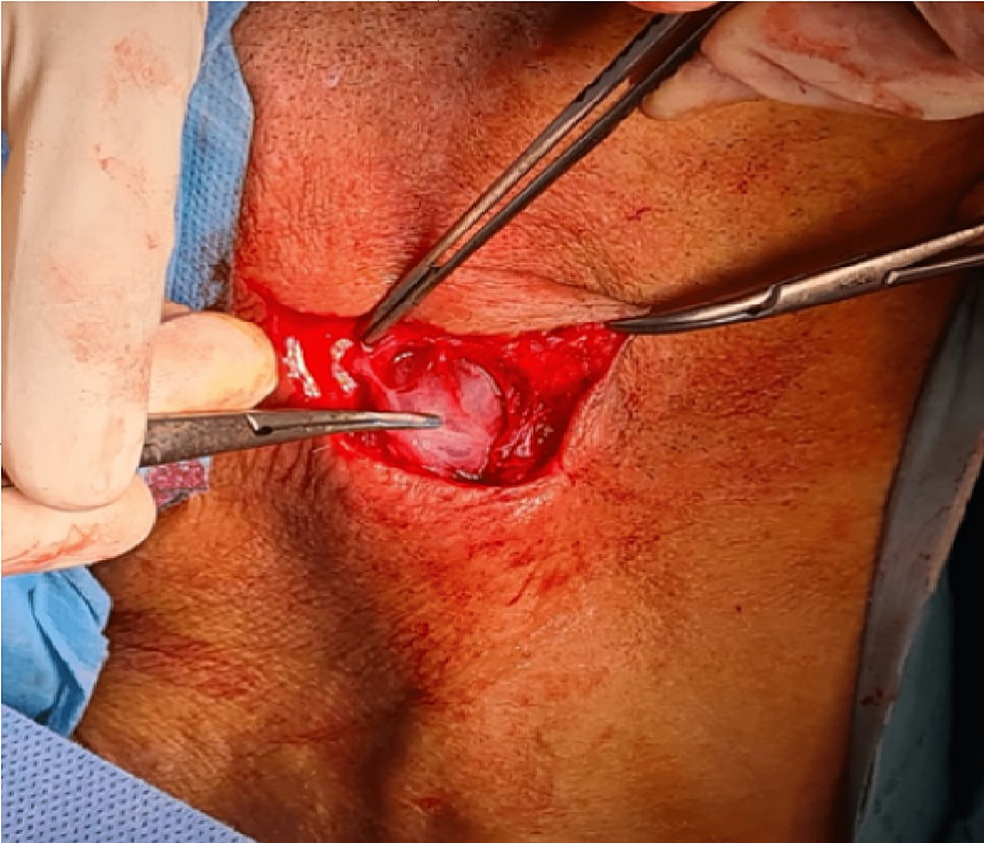
Incision centered on the adenopathy Photo from Cheikh Khalifa International University Hospital ENT department

While the biopsy is performed when the dissection is impossible due to adhesions, those adhesions are due to the infectious or inflammatory preganglionic component. However, according to the literature, biopsy should be prohibited because it exposes the patient to a high risk of fistulization [11]. It also represents a risk of capsular rupture in the context of aggravating metastatic adenopathies as well as the prognosis [11]. Adenectomy is an essential procedure, especially in the event of doubtful cytology or negative bacteriological examination. It allows the entire pathological hypertrophied lymph node to be excised and is recommended by most authors [11] (Figure 6).

**Figure 7:**
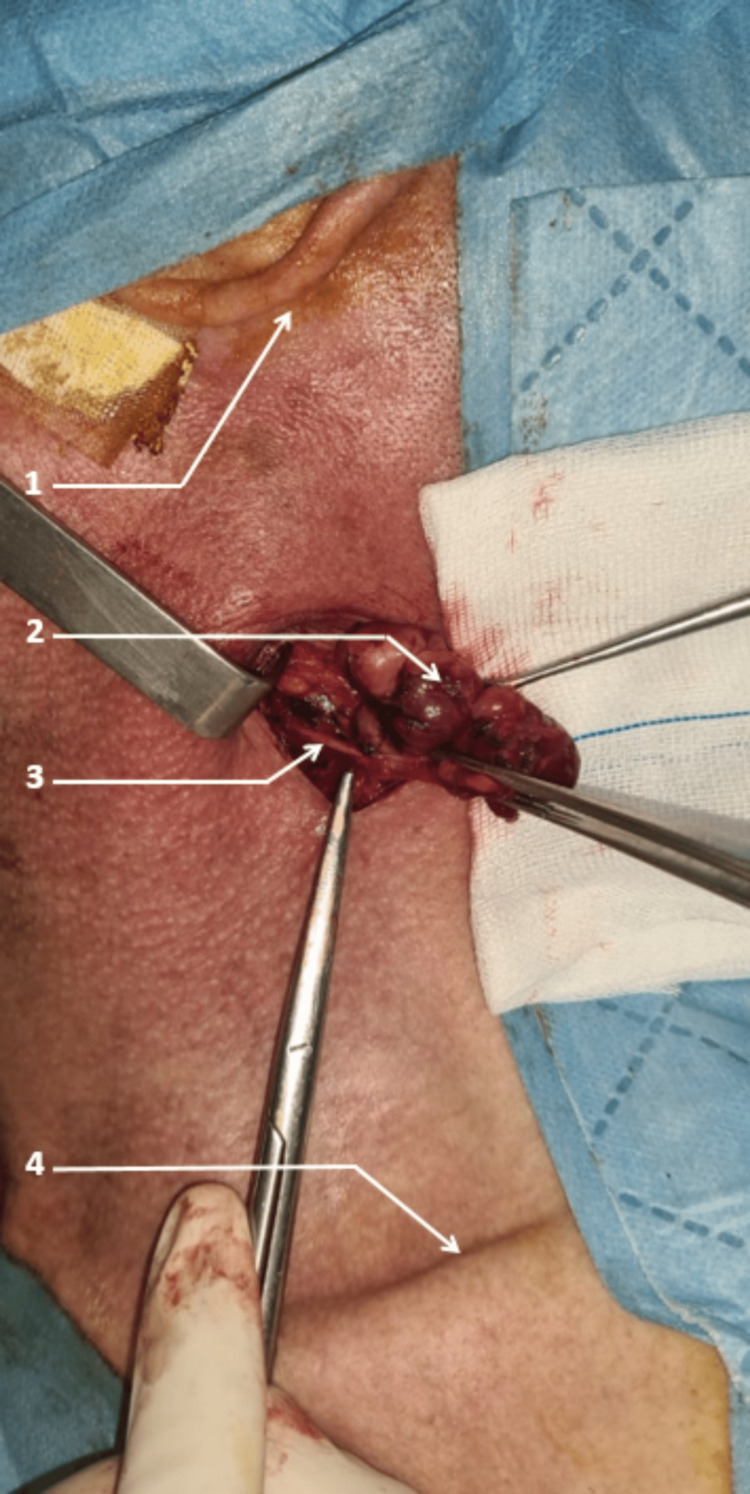
Polyadenopathy adherent to the spinal nerve 1 : Earlobe 2 : Polyadenopathy 3 : Spinal nerve 4 : Clavicle Photo from Cheikh Khalifa International University Hospital ENT department

Lymphadenectomy, on the other hand, corresponds to an unsystematic excision, more or less wide depending on the territory, of several adenopathies and lymph node tissue around them [11] (Figure 7).

In our study, the use of adenectomy (54 patients, i.e., 51.9%) was higher than reported in other studies (47% and 41.5%) [26, 43]. While the use of dissection or lymphadenectomy (24 patients i.e., 23.1%) was lower than the rate of those same studies (47% and 47.4%). Finally, in our study, the lymph node drainage rate was 13.5% (i.e., 14 patients), which is higher than what was observed in those studies (4% and 7.5%).

In our study, the relapse rate was 10.6% (i.e., 11 patients), the treatment failure rate was 3.8% (i.e., four patients), while the paradoxical reaction only affected 2.9% (i.e., three patients). Those patients had a simple biopsy. As a result, the use of a broader gesture makes it possible to give better results with a better rate of healing and fewer complications. Our results are similar to two studies carried out in Morocco, at the University Hospital Centre (CHU) of Fez and CHU of Marrakech, which report relapse rates ranging from 7% to 17% [44].

There is little Evidence-Based Medicine (EBM) in favor of the indications of the different surgical techniques proposed, hence the need for multiple studies in this direction to better codify the therapeutic indications. However, our study presents a selection bias because it was conducted within the ENT Department, which does not reflect the sum total reality of the population affected by LNTB. To obtain more complete results, it would be wise to have a study grouping together all patients with cervical LNTB in the ENT and Pulmonology departments.

In the end, our study supports and reinforces the theory of the association of medical treatment associated with surgical treatment [43,45-46]. Indeed, medical treatment remains essential in the management of cervical LNTB. But certain situations, such as the appearance of a fistula or an abscess, immediately impose the need for surgical management.

## Conclusions

TB still represents a real public health problem in Morocco, involving many actors. Diagnosis and treatment constitute a challenge requiring multidisciplinary care. In our study, cervicotomy for diagnostic and therapeutic purposes associated with a histological study was the key to diagnosis.

Anti-bacillary therapy is the gold standard treatment for LNTB. However, surgery retains all its interest, not only for the diagnosis of TB but also for carrying out first-line therapeutic procedures. Surgery also shows great promise in situations where medical treatment proves to be insufficient, particularly in the event of treatment failure, relapse, or resistance to standard medical treatment.
